# Systemic inflammation and the risk of adverse clinical outcomes in heart failure with preserved ejection fraction

**DOI:** 10.3389/fcvm.2026.1872310

**Published:** 2026-08-28

**Authors:** Chi Nguyen, Guanghao Zhang, Prashanth Iyer, Amanda Ackermann, Jeffrey R. Skaar, Wing Chow, Radha Ryali, Josephine Harrington

**Affiliations:** 1 Novo Nordisk Inc., Plainsboro, NJ, United States; 2 University of Colorado Health, Aurora, CO, United States

**Keywords:** adverse clinical outcomes, heart failure, heart failure with preserved ejection fraction (HFpEF), high-sensitivity C-reactive protein (hsCRP), systemic inflammation

## Abstract

**Introduction:**

Systemic inflammation is implicated in the pathogenesis of cardiovascular disease, including heart failure (HF). We examined the association between systemic inflammation, indicated by elevated high-sensitivity C-reactive protein (hsCRP), and adverse clinical outcomes in individuals with HF with preserved ejection fraction (HFpEF).

**Methods:**

This retrospective cohort study identified individuals with HFpEF from the Komodo Healthcare Map™ database (01/01/2016 to 12/31/2023) using a validated algorithm. Systemic inflammation status was established by hsCRP testing: with systemic inflammation if ≥1 hsCRP value of 2-10 mg/L and without systemic inflammation if all hsCRP values <2 mg/L. The relationship between hsCRP and risk for HF events, including all-cause mortality, HF hospitalization and urgent HF visits was examined using Cox proportional hazards models with multivariable adjustment.

**Results:**

A total of 11,015 individuals with HFpEF and a qualifying hsCRP measurement were identified (mean age 66.4 years; 44% with systemic inflammation). The incidence rate of the composite HF endpoint was 103.2 per 1,000 person-years for those with systemic inflammation versus 83.3 for those without. Systemic inflammation was associated with a 21% increased risk of the composite HF endpoint (HR 1.21, 95% CI 1.11-1.31). Systemic inflammation was associated with increased risk of HF hospitalization (HR 1.17, 95% CI 1.03-1.33) and urgent HF visits (HR 1.21, 95% CI 1.08-1.35) but not all-cause mortality (HR 1.14, 95% CI 0.98-1.32).

**Conclusion:**

Systemic inflammation was associated with increased risk of future adverse CV-related outcomes in individuals with HFpEF, highlighting unmet clinical needs for this population and the potential to optimize their care.

## 1 Introduction

Approximately 6.7 million adults in the US live with heart failure (HF), and this population is projected to exceed 8 million by 2030 (1). HF is associated with significant morbidity and mortality. One in every eight deaths in the US is attributable to HF, and it is the primary cause of nearly one million hospitalizations annually (2).

Individuals with HF are classified based on left ventricular ejection fraction (LVEF) as HF with reduced ejection fraction (HFrEF, LVEF ≤40%), mildly reduced ejection fraction (HFmrEF, LVEF 41%–49%), and HF with preserved ejection fraction (HFpEF, LVEF ≥50%) (3). While there are multiple therapeutic options for HFrEF, there are limited effective therapies for HFpEF/HFmrEF (3). Although sacubitril/valsartan and sodium-glucose cotransporter-2 (SGLT2) inhibitors (empagliflozin and dapagliflozin) are approved for HFpEF, therapeutic options mainly focus on managing comorbidities and using diuretics to alleviate symptoms, and significant unmet need remains for people with HFpEF or HFmrEF (3, 4).

Systemic inflammation plays an established role in cardiovascular disease, particularly atherosclerotic cardiovascular disease (ASCVD), where many studies have demonstrated that cardiovascular inflammation is associated with poor clinical outcomes (5). The role of systemic inflammation in HF is less established, but increasing data suggest that systemic inflammation may drive HF (6), particularly HFpEF. In HFpEF, systemic inflammation may drive myocardial remodelling and increase diastolic left ventricular stiffness by increasing collagen deposition in the myocardial interstitium and reducing elasticity of cardiomyocytes (7). Multiple studies have examined the role of the pro-inflammatory cytokine interleukin-6 (IL-6), and its downstream biomarker, C-reactive protein (often measured by a high-sensitivity test; hsCRP) in cardiovascular disease. In the Biology Study to Tailored Treatment in Chronic Heart Failure (BIOSTAT-CHF) trial, elevated levels of IL-6 were detected in 56% of individuals with HF and were independently associated with higher risks of HF hospitalization and all-cause mortality (8). Elevated CRP or hsCRP as markers of systemic inflammation have been independently linked to two-fold increased cardiovascular mortality in individuals with HF (9, 10). While prior analyses, including *post-hoc* analyses of clinical trials and meta-analyses, have examined systemic inflammation in HFpEF, no large-scale real-world observational study has specifically characterized their association, and routine hsCRP or IL-6 testing is not currently supported by guidelines for HF (11). Therefore, due to the lack of studies and paucity of hsCRP testing, the clinical burden of illness associated with systemic inflammation in HFpEF remains unclear.

This study explored the association between systemic inflammation, as indicated by hsCRP levels, and the risk of experiencing clinical HF outcomes, including HF hospitalization, urgent HF visits, and all-cause mortality, in individuals with HFpEF identified in a large US administrative claims and laboratory database.

## 2 Materials and methods

### 2.1 Study design and population

This retrospective cohort study utilized the Komodo Healthcare Map™ database, which includes de-identified administrative medical and pharmacy claims linked with clinical and laboratory measurements for a nationally representative US population (12) between 01/01/2016 and 12/31/2023. Individuals with HF were identified through at least one inpatient claim or two outpatient claims with an International Classification of Diseases, 10th Revision, Clinical Modification (ICD-10-CM) diagnosis of HF (I50x). The cohort comprised both incident (newly diagnosed) and prevalent (existing) cases of HF, with the date of the initial diagnosis during the identification period designated as the index date.

Since LVEF data were not available and more than 40% of individuals with HF identified by ICD-10-CM codes had unspecified HF (vs. systolic or diastolic HF), a validated algorithm was employed to predict LVEF and classify HF. The Desai et al. algorithm was developed to classify HF using Medicare administrative claims (13) and later validated using predominantly commercial insurance administrative claims from MarketScan data (14). The algorithm uses demographics, HF-related diagnoses (ICD-10-CM codes indicating systolic, diastolic, left, rheumatic, hypertensive, or unspecified HF), number of HF hospitalizations, whether HF is diagnosed in inpatient or outpatient setting, history of implantable cardioverter defibrillator, cardiac resynchronization therapy, left ventricular assist device, HF-related medication use, and comorbid conditions for HF classification. The model has a positive predictive value (PPV) of 0.84 and sensitivity of 0.97 for HFpEF. We used this validated algorithm to classify HF and conducted multiple sensitivity analyses to verify the classification.

Included individuals were required to have at least one eligible hsCRP test result within 1 year before or after the index date to determine their systemic inflammation status. Eligible individuals were classified as either having systemic inflammation (at least one hsCRP value of 2–10 mg/L) or without systemic inflammation (all hsCRP values <2 mg/L). hsCRP tests with values >10 mg/L and tests taken during acute infection, shortly after acute events, or while taking antimicrobial medications or corticosteroids were excluded to avoid the assessment of elevated hsCRP levels impacted by acute conditions.

**Figure 1:**
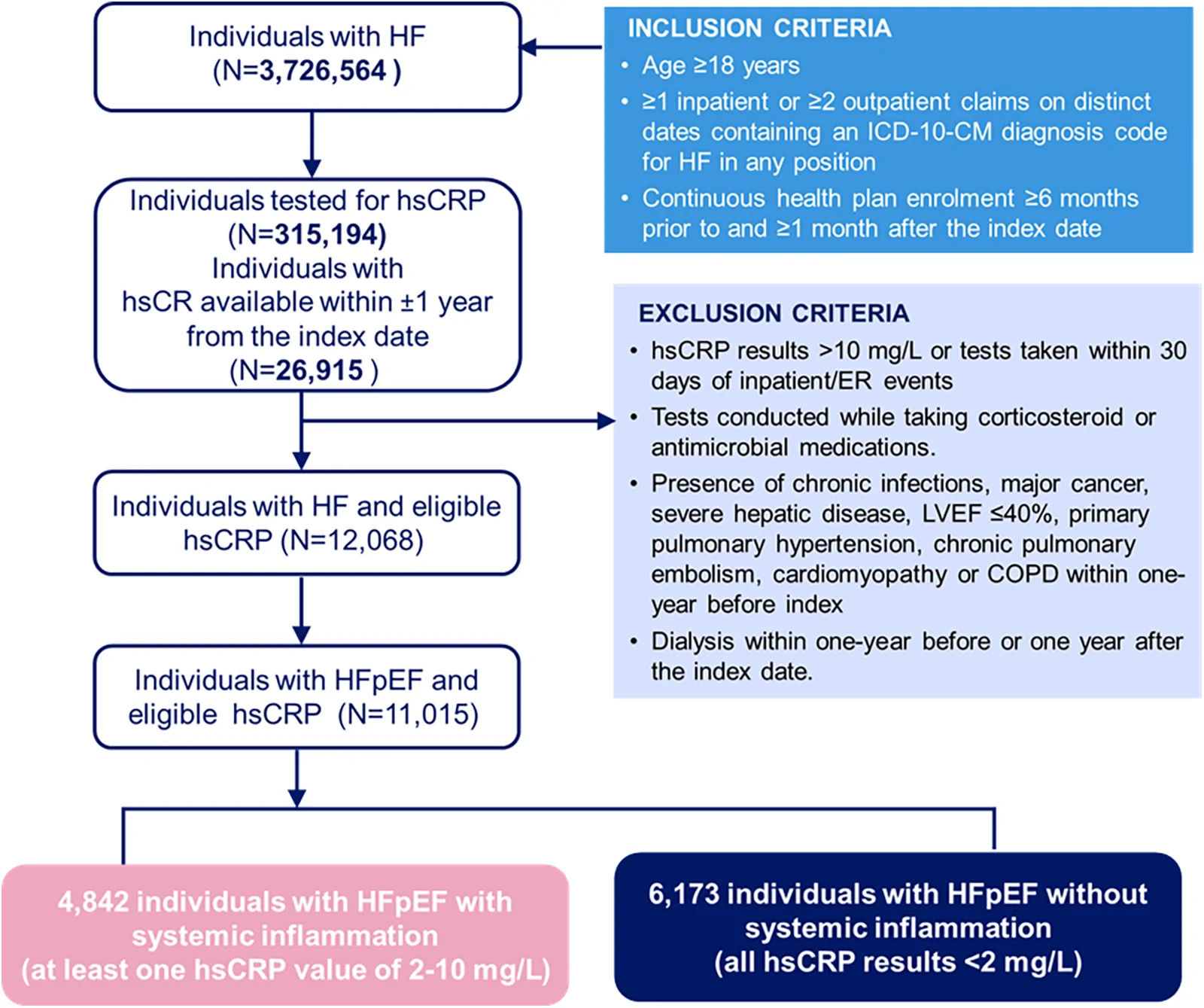
Selection of the study population. COPD, chronic obstructive pulmonary disease; ER, emergency room; HF, heart failure; HFpEF, heart failure with preserved ejection fraction; hsCRP, high-sensitivity C-reactive protein; ICD-10-CM, international classification of diseases, 10th revision, clinical modification; LVEF, left ventricular ejection fraction.

Individuals were excluded from the study if they had severe hepatic disease (hepatic encephalopathy, ascites, or hepatic cirrhosis), chronic infectious disease (hepatitis, HIV, or tuberculosis), cancer (except skin cancer) or required kidney dialysis during the period of hsCRP assessment (1 year before or 1 year after the index date), as these may impact hsCRP levels. Individuals with primary pulmonary hypertension, severe chronic obstructive pulmonary disease (COPD), or acute myocarditis during the baseline period were also excluded. An overview of the HFpEF cohort selection criteria is presented in Figure 1.

### 2.2 Outcome measures

The primary outcome measure was a composite HF endpoint consisting of HF hospitalization, urgent HF visits, and all-cause mortality. HF hospitalization was defined as inpatient admission with a primary diagnosis of HF. Urgent HF visit was defined as emergency room or outpatient visit with a primary diagnosis of HF and at least one of the following treatments or procedures specifically for HF: (a) intravenous diuretic or vasoactive agent or (b) mechanical or surgical intervention, such as mechanical circulatory support or mechanical fluid removal. All-cause mortality data were sourced from multiple databases, including the Death Master File (DMF) provided by the Social Security Administration (SSA), inpatient discharge status, and third-party obituary data. Specific cause of death data were not available. An alternative definition of HF hospitalization and urgent HF visit was used that included inpatient admission or urgent visit with a diagnosis of HF in any position, instead of restricted to the primary diagnosis.

### 2.3 Statistical analysis

Descriptive statistics were reported to summarize the baseline characteristics of the HFpEF population, including means, standard deviations, medians, and interquartile ranges for continuous variables, as well as counts and percentages for categorical variables. The statistical differences between cohorts (i.e., HFpEF with and without systemic inflammation) were evaluated using Chi-square tests or Fisher's exact tests for categorical variables and *t*-tests or Wilcoxon rank-sum tests for continuous variables.

Kaplan–Meier survival curves were constructed to estimate the time from the index date to the first occurrence of a HF event (HF hospitalization, urgent HF visit, or all-cause mortality). The log-rank test was utilized to compare the survival distributions between the two cohorts. The incidence rate of HF endpoints was estimated as the number of individuals who experienced a new event (e.g., first HF endpoint following the index date) divided by the total time-at-risk for all individuals and was reported as the number of events per 1,000 person-years during the follow-up period.

To assess the association between systemic inflammation and the risk of HF events, a Cox proportional hazards model was used. In this model, the time to the first HF event was the dependent variable, while systemic inflammation status and relevant baseline characteristics (including age, gender, race/ethnicity, insurance type, comorbidities, and baseline medication use) served as independent variables.

All analyses were conducted using R statistical software and a two-tailed significance level of *P* < 0.05 was adopted to determine statistical significance.

### 2.4 Sensitivity and representativeness analysis

A sensitivity analysis was conducted using ICD-10-CM codes for HF classification, identifying individuals with HFpEF using the ICD-10-CM code for diastolic HF (I50.3) instead of using the Desai et al. algorithm (13). The representativeness of the study population was evaluated by comparing the baseline characteristics of eligible individuals with HFpEF who were tested for hsCRP, individuals with HFpEF not tested for hsCRP, and the whole study population.

## 3 Results

### 3.1 Cohort demographics and baseline characteristics

Out of the 3,727,042 individuals with HF, 8.5% underwent testing for hsCRP during the study period from 2016 to 2023, and 2.0% had hsCRP test results available within the database. After applying the inclusion and exclusion criteria, a total of 12,068 individuals with HF and at least one eligible hsCRP test result within ±1 years of the index date were identified. Of these, 11,015 were classified as having HFpEF using the Desai algorithm and 44.0% (*N* = 4,842) exhibited systemic inflammation (Figure 1).

The mean age of the population was 66.4 ± 12.4 years, with equal representation of females and males (48.7% and 48.2%, respectively, with 3.1% missing data). Among the included individuals, 53.0% were White, 17.7% as Hispanic/Latino, 13.6% as Black/African American, 4.8% as Asian/Pacific Islander, 4.1% from other racial/ethnic groups, and 6.8% with missing data. A total of 55.9% were on Medicare Advantage or Medicare fee-for-service, and 12.6% were on Medicaid (Table 1).

**Table 1:** Demographics and clinical characteristics.

Variables	HFpEF with systemic inflammation	HFpEF without systemic inflammation	Total	*P* value
*N*	4,842	6,173	11,015	
Age at index, mean (SD)	65.9 (12.4)	66.9 (12.5)	66.4 (12.4)	*P* < 0.01
Gender				*P* < 0.01
Female	2,482 (51.3%)	2,880 (46.7%)	5,362 (48.7%)	
Male	2,213 (45.7%)	3,094 (50.1%)	5,307 (48.2%)	
Missing	147 (3.0%)	199 (3.2%)	346 (3.1%)	
Race/Ethnicity				*P* < 0.01
White	2,483 (51.3%)	3,358 (54.4%)	5,841 (53.0%)	
Hispanic or Latino	881 (18.2%)	1,069 (17.3%)	1,950 (17.7%)	
Black or AfricanAmerican	742 (15.3%)	753 (12.2%)	1,495 (13.6%)	
Asian or PacificIslander	180 (3.7%)	351 (5.7%)	531 (4.8%)	
Other	186 (3.8%)	269 (4.4%)	455 (4.1%)	
Missing	370 (7.6%)	373 (6.0%)	743 (6.8%)	
Region				*P* < 0.01
Northeast	1,230 (25.4%)	1,824 (29.6%)	3,054 (27.7%)	
North Central	562 (11.6%)	672 (10.9%)	1,234 (11.2%)	
South	1,752 (36.2%)	1,841 (29.8%)	3,593 (32.6%)	
West	1,296 (26.8%)	1,827 (29.6%)	3,123 (28.4%)	
Missing	2 (0.04%)	9 (0.2%)	11 (0.1%)	
Insurance				*P* = 0.11
Commercial	1,553 (32.1%)	1,920 (31.1%)	3,473 (31.6%)	
Medicaid	638 (13.2%)	746 (12.1%)	1,384 (12.6%)	
Medicare	2,648 (54.7%)	3,505 (56.8%)	6,153 (55.9%)	
Missing	3 (0.06%)	2 (0.03%)	5 (0.05%)	
QCI				*P* < 0.01
Mean (SD)	1.06 (1.3)	0.98 (1.3)	1.01 (1.3)	
Categorical QCI				*P* < 0.01
0	2,237 (46.2%)	3,066 (49.7%)	5,303 (48.1%)	
1–2	1,956 (40.4%)	2,359 (38.2%)	4,315 (39.2%)	
3–4	559 (11.5%)	652 (10.6%)	1,211 (11.0%)	
5+	90 (1.9%)	96 (1.6%)	186 (1.7%)	

*P* values show the statistical differences between the two cohorts (HFpEF with and without systemic inflammation) evaluated using Chi-square tests or Fisher's exact test for categorical variables and *t*-test or Wilcoxon rank-sum tests for continuous variables. HFpEF, heart failure with preserved ejection fraction; SD, standard deviation; QCI, Quan–Charlson Comorbidity Index.

A comparison of the baseline clinical and demographic characteristics among eligible individuals with HFpEF who were not tested for hsCRP (*N* = 1,995,610) and those who were tested for hsCRP (*N* = 185,637), showed that individuals tested for hsCRP were younger (64.5 vs. 67.5 years), more likely to have commercial insurance health plans (27.6% vs. 21.5%), and had a slightly higher Quan–Charlson Comorbidity Index (15) (QCI; 1.23 vs. 1.18). A detailed overview of the baseline characteristics of these two groups as well as all eligible individuals (*N* = 2,181,247) and those in the final study population (*N* = 11,015) is presented in Supplementary Table S1.

**Figure 2:**
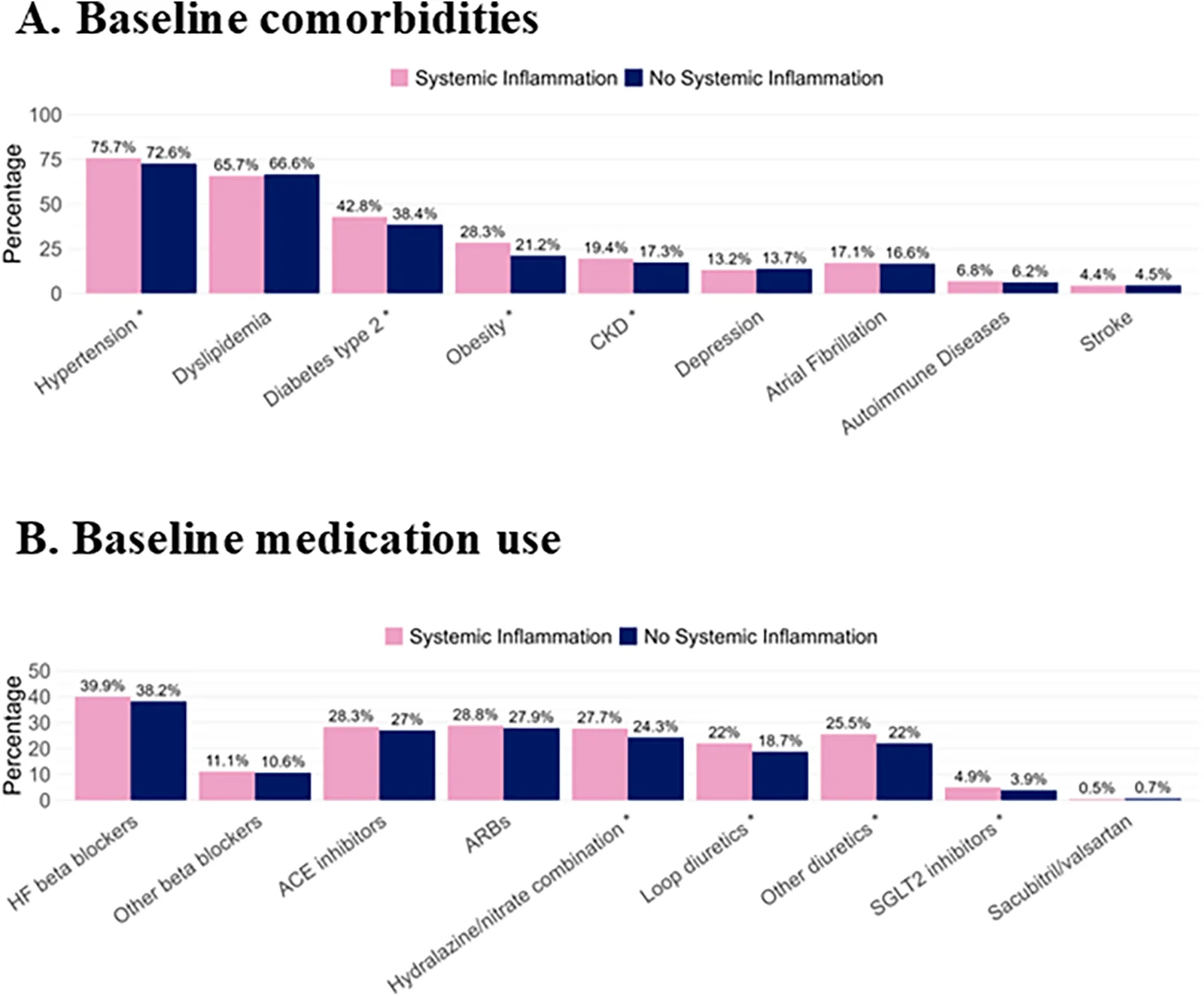
Baseline comorbidities **(A)** and medication use **(B)**. Numbers are percentages of all included individuals. *P*-values were estimated using *t*-test/Wilcoxon test for continuous variables and Chi-square test for categorical variables. *P < 0.05.

Individuals with systemic inflammation were significantly more likely to be female (51.3% vs. 46.7%) and had significantly more comorbidities than those without systemic inflammation, including hypertension (75.7% vs. 72.6%), type 2 diabetes (42.8% vs. 38.4%), obesity (28.3% vs. 21.2%) and chronic kidney disease (19.4% vs. 17.3%) (all *P* < 0.05; Figure 2A). During 6 months prior to HF diagnosis, individuals with systemic inflammation were more often prescribed loop diuretics (22.0% vs. 18.6%) and other diuretics (25.5% vs. 22.0%), SGLT2 inhibitors (4.9% vs. 3.9%), and hydralazine/nitrate combinations (27.7% vs. 24.3%) (all *P* < 0.05; Figure 2B).

### 3.2 Incidence rates of HF outcomes

Over a mean follow-up time of 33.2 months, the incidence rate of the composite HF endpoint identified by HF diagnosis in primary position on claims was 103.2 (95% CI 97.3–109.3) per 1,000 person-years for those with systemic inflammation and 83.3 (95% CI 78.7–88.0) for those without. For the individual clinical outcomes, the incidence rate for HF hospitalization was 41.2 (95% CI 37.7–45.0) vs. 33.6 (95% CI 30.9–36.6), for urgent HF visits 55.6 (95% CI 51.4–60.0) vs. 43.4 (95% CI 40.2–46.8), and for all-cause mortality 28.0 (95% CI 25.2–31.0) vs. 25.0 (95% CI 22.7–27.5) in individuals with and without systemic inflammation, respectively (Table 2).

**Table 2:** Incidence rates of HF events in individuals with or without HFpEF.

*N*	HFpEF without systemic inflammation	HFpEF with systemic inflammation	Total
	6,173	4,842	11,015
Main analysis—HF as a principal diagnosis			
Length of follow-up (months)	33.3	33.1	33.2
HF hospitalization (HF diagnosis code in primary position)	33.6 (30.9, 36.6)	41.2 (37.7, 45.0)	36.9 (34.7, 39.2)
Urgent HF visits (HF diagnosis code in primary position)	43.4 (40.2, 46.8)	55.6 (51.4, 60.0)	48.7 (46.1, 51.4)
All-cause mortality	25.0 (22.7, 27.5)	28.0 (25.2, 31.0)	26.3 (24.5, 28.2)
Composite HF endpoint	83.3 (78.7, 88.0)	103.2 (97.3, 109.3)	91.8 (88.2, 95.6)
Alternative definition—HF diagnosis in any position in claims			
HF hospitalization (HF diagnosis code in any position)	90.8 (86.0, 95.9)	104.4 (98.5, 110.6)	96.7 (92.1, 100.6)
Urgent HF visits (HF diagnosis code in any position)	68.7 (64.5, 73.0)	84.2 (78.9, 89.7)	75.3 (72.0, 78.8)
All-cause mortality	25.0 (22.7, 27.5)	28.0 (25.2, 31.0)	26.3 (24.5, 28.2)
Composite HF endpoint	145.0 (138.6, 151.6)	178.6 (170.3, 187.1)	159.2 (154.1, 164.5)

Incidence rates with 95% CI are listed. CI, confidence interval; HF, heart failure; HFpEF, heart failure with preserved ejection fraction.

Using HF diagnosis code in any position to identify HF outcomes yielded similar findings, with higher incidence rates of all-cause hospitalization, HF-related hospitalizations and urgent HF visits in individuals with systemic inflammation compared to those without (Table 2).

**Figure 3:**
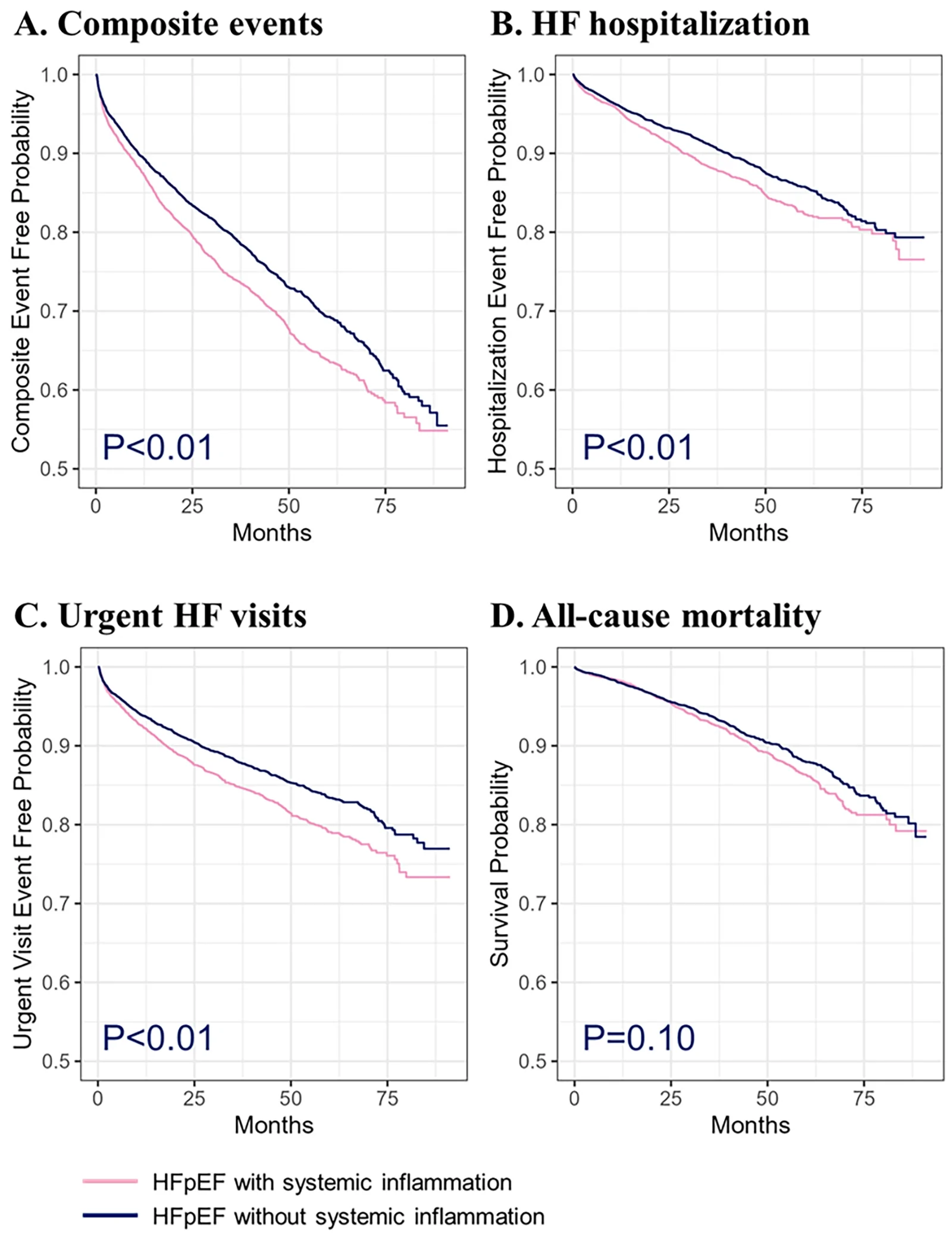
Time from HF diagnosis to the first composite HF event **(A)** or its components HF hospitalization **(B)**, urgent HF visit **(C)**, and all–cause mortality **(D)** in individuals with HFpEF. Kaplan–Meier survival curves were constructed to estimate the time from the index date to the first occurrence of specific heart failure events. The log-rank test was utilized to compare the survival distributions between the two cohorts.

To understand the time to event for the composite endpoint and its components, Kaplan–Meier analyses and log-rank tests were conducted. Individuals with systemic inflammation experienced significantly higher incidences of the HF composite endpoint, HF hospitalization and urgent HF visits than those without systemic inflammation (all *P* < 0.01; Figures 3A–C). No significant difference was observed for time to all-cause mortality (*P* = 0.10; Figure 3D).

### 3.3 Adjusted risks of HF outcomes

After controlling baseline characteristics using a Cox proportional hazards model, systemic inflammation was significantly associated with a 21% increase in the risk of experiencing the composite HF endpoint (HR 1.21, 95% CI 1.11–1.31, *P* < 0.01). When examining HF events individually, systemic inflammation was significantly associated with higher risk of both HF hospitalization (HR 1.17, 95% CI 1.03–1.33, *P* = 0.01) and urgent HF visits (HR 1.21, 95% CI 1.08–1.35, *P* < 0.01), but not with overall mortality (HR 1.14, 95% CI 0.98–1.32, *P* = 0.09) (Table 3).

**Table 3:** Factors associated with the HF composite endpoint or its components HF hospitalization, urgent HF visits and all–cause mortality.

Variable	HF composite endpoint		HF hospitalization		Urgent HF visits		All-cause mortality	
	HR (95% CI)	*P* value	HR (95% CI)	*P* value	HR (95% CI)	*P* value	HR (95% CI)	*P* value
Systemic inflammation								
No	Reference							
Yes	1.21 (1.11, 1.31)	<0.01	1.17 (1.03, 1.33)	0.01	1.21 (1.08, 1.35)	<0.01	1.14 (0.98, 1.32)	0.09
Age								
18–44	Reference							
45–54	1.22 (0.9, 1.66)	0.19	1.26 (0.79, 2)	0.34	1.16 (0.81, 1.65)	0.42	0.86 (0.39, 1.89)	0.71
55–64	1.27 (0.96, 1.68)	0.1	1.17 (0.76, 1.81)	0.47	1.11 (0.79, 1.54)	0.55	1.21 (0.6, 2.44)	0.59
65–74	1.13 (0.84, 1.52)	0.42	1.26 (0.8, 1.99)	0.33	0.85 (0.6, 1.22)	0.38	1.42 (0.7, 2.89)	0.34
75–84	1.75 (1.3, 2.35)	<0.01	1.91 (1.21, 3)	0.01	1.17 (0.82, 1.67)	0.38	3.04 (1.5, 6.15)	<0.01
≥85	2.8 (1.98, 3.97)	<0.01	3.3 (1.94, 5.64)	<0.01	1.47 (0.93, 2.32)	0.1	7.78 (3.67, 16.5)	<0.01
Gender								
Female	Reference							
Male	1.1 (1.01, 1.19)	0.04	1.09 (0.96, 1.25)	0.18	0.99 (0.88, 1.11)	0.9	1.44 (1.24, 1.67)	<0.01
Race/Ethnicity								
White	Reference							
Hispanic or Latino	0.84 (0.75, 0.95)	<0.01	0.98 (0.82, 1.16)	0.78	0.88 (0.75, 1.03)	0.12	0.61 (0.49, 0.76)	<0.01
Black or African American	1.08 (0.95, 1.22)	0.25	1.12 (0.94, 1.35)	0.21	1.22 (1.04, 1.44)	0.01	0.78 (0.62, 0.99)	0.04
Asian or Pacific Islander	0.69 (0.55, 0.88)	<0.01	0.77 (0.54, 1.1)	0.15	0.85 (0.63, 1.14)	0.27	0.48 (0.28, 0.81)	0.01
Other	0.8 (0.63, 1.02)	0.08	0.89 (0.62, 1.28)	0.52	0.85 (0.62, 1.15)	0.29	0.55 (0.32, 0.97)	0.04
Region								
North East	Reference							
North Central	1.01 (0.87, 1.16)	0.92	0.96 (0.77, 1.19)	0.68	1.03 (0.84, 1.25)	0.79	1.28 (1.02, 1.6)	0.03
South	1 (0.89, 1.11)	0.95	0.9 (0.76, 1.07)	0.23	1.02 (0.87, 1.18)	0.82	1.14 (0.94, 1.37)	0.19
West	1.12 (1, 1.25)	0.04	1.03 (0.87, 1.22)	0.72	1.26 (1.09, 1.47)	<0.01	0.88 (0.72, 1.08)	0.21
Insurance								
Commercial	Reference							
Medicaid	1.31 (1.11, 1.55)	<0.01	1.49 (1.16, 1.91)	<0.01	1.33 (1.08, 1.63)	0.01	1.15 (0.74, 1.79)	0.53
Medicare	1.29 (1.11, 1.5)	<0.01	1.21 (0.95, 1.53)	0.12	1.22 (1, 1.48)	0.05	2.08 (1.5, 2.89)	<0.01
QCI								
0	Reference							
1–2	1.29 (1.17, 1.42)	<0.01	1.4 (1.2, 1.63)	<0.01	1.14 (1, 1.29)	0.05	1.41 (1.18, 1.68)	<0.01
3–4	1.48 (1.3, 1.69)	<0.01	1.77 (1.45, 2.15)	<0.01	1.29 (1.08, 1.55)	0.01	1.67 (1.33, 2.09)	<0.01
5+	1.54 (1.2, 1.98)	<0.01	1.72 (1.18, 2.5)	<0.01	0.93 (0.62, 1.39)	0.72	2.16 (1.47, 3.17)	<0.01
Baseline conditions								
Atrial fibrillation	1.33 (1.2, 1.48)	<0.01	1.29 (1.1, 1.51)	<0.01	1.49 (1.29, 1.71)	<0.01	1.22 (1.03, 1.46)	0.02
Depression	1 (0.89, 1.14)	0.94	1.13 (0.95, 1.35)	0.17	0.87 (0.73, 1.04)	0.13	1.02 (0.83, 1.27)	0.83
Dyslipidemia	0.85 (0.77, 0.94)	<0.01	0.91 (0.79, 1.06)	0.24	0.85 (0.74, 0.97)	0.01	0.83 (0.7, 0.98)	0.03
Stroke	1.45 (1.23, 1.72)	<0.01	1.17 (0.9, 1.53)	0.25	1.36 (1.07, 1.73)	0.01	1.59 (1.22, 2.09)	<0.01
Hypertension	1.11 (0.98, 1.25)	0.1	1.03 (0.85, 1.25)	0.73	1.08 (0.92, 1.27)	0.34	1.2 (0.96, 1.49)	0.11
Hospitalization during 6 months prior to the index date	1.2 (1.07, 1.35)	<0.01	1.3 (1.1, 1.54)	<0.01	0.96 (0.82, 1.14)	0.67	1.36 (1.12, 1.65)	<0.01
Baseline Medication								
Anti-inflammatory andimmunosuppressant medications	0.95 (0.8, 1.14)	0.61	0.98 (0.76, 1.27)	0.89	1.11 (0.88, 1.39)	0.38	0.92 (0.67, 1.26)	0.61
ACE inhibitors	1 (0.91, 1.11)	0.99	1.04 (0.9, 1.21)	0.6	1.06 (0.92, 1.21)	0.42	0.9 (0.75, 1.07)	0.23
MRAs	1.15 (0.97, 1.37)	0.11	1.14 (0.88, 1.47)	0.32	1.22 (0.98, 1.51)	0.08	1.16 (0.85, 1.58)	0.35
ARBs	0.93 (0.84, 1.03)	0.18	0.96 (0.82, 1.12)	0.62	0.98 (0.85, 1.13)	0.76	0.8 (0.67, 0.96)	0.02
HF beta blockers	1.07 (0.98, 1.17)	0.15	1.14 (0.99, 1.31)	0.06	1.11 (0.98, 1.25)	0,1	0.96 (0.82, 1.12)	0.59
Other beta blockers	1.12 (0.99, 1.27)	0.08	1.04 (0.85, 1.26)	0.73	1.11 (0.93, 1.32)	0.23	1.06 (0.85, 1.33)	0.59
Loop diuretics	1.77 (1.61, 1.94)	<0.01	1.8 (1.56, 2.07)	<0.01	2.1 (1.86, 2.38)	<0.01	1.39 (1.18, 1.64)	<0.01
Other diuretics	0.92 (0.79, 1.07)	0.26	0.82 (0.66, 1.02)	0.07	0.93 (0.77, 1.14)	0.5	1.12 (0.87, 1.45)	0.37
Hydralazine/nitrate combination	1.17 (1.02, 1.35)	0.03	1.39 (1.14, 1.71)	<0.01	1.22 (1.01, 1.47)	0.04	0.98 (0.76, 1.25)	0.85
Digoxin	1.33 (1.05, 1.68)	0.02	1.09 (0.76, 1.59)	0.63	1.53 (1.15, 2.05)	<0.01	1.17 (0.78, 1.77)	0.44
Sacubitril/valsartan or ivabradine	1.37 (0.84, 2.26)	0.21	2.64 (1.48, 4.72)	<0.01	1.38 (0.75, 2.51)	0.3	0.55 (0.14, 2.24)	0.41
SGLT2 inhibitors	1.02 (0.81, 1.29)	0.84	0.87 (0.6, 1.26)	0.47	1.08 (0.81, 1.44)	0.61	1.08 (0.67, 1.74)	0.75

Cox proportional hazards model was applied to evaluate association of HF events and baseline characteristics. HR and 95% CI as well as the associated *P*-values are listed for the composite HF endpoint and its three components HF hospitalization, urgent HF visits and all-cause mortality. ACE, angiotensin converting enzyme; ARB, angiotensin receptor blocker; CI, confidence interval; HF, heart failure; HR, hazard ratio; MRA, mineralocorticoid receptor antagonist; QCI, Quan–Charlson Comorbidity Index; SGLT2, sodium-glucose cotransporter-2.

The Cox proportional hazards model identified several baseline demographics that were associated with an increased risk of experiencing a composite HF event. These factors included being over 75 years old, male sex, residing in the Western region, and being enrolled in Medicare or Medicaid insurance. Higher comorbidity index as measured by QCI also indicated higher risks of composite HF events: HR 1.29 with 1–2 comorbidities (95% CI 1.17–1.42, *P* < 0.01), HR 1.48 with 3–4 comorbidities (95% CI 1.30–1.69, *P* < 0.01), and HR 1.54 with 5 or more comorbidities (95% CI 1.20–1.98, *P* < 0.01). Additionally, the presence of specific baseline conditions such as atrial fibrillation (HR 1.33, 95% CI 1.20–1.48, *P* < 0.01) and stroke (HR 1.45, 95% CI 1.23–1.72, *P* < 0.01) significantly correlated with incidence of the HF composite endpoint. Furthermore, baseline medication use of loop diuretics (HR 1.77, 95% CI 1.61–1.94, *P* < 0.01), hydralazine/nitrate combinations (HR 1.17, 95% CI 1.02–1.35, *P* = 0.03), and digoxin (HR 1.33, 95% CI 1.05–1.68, *P* = 0.02) were associated with increased risk. Details of each of the components of the endpoints are also presented in Table 3.

### 3.4 Sensitivity and representativeness analysis

The sensitivity analysis using ICD-10-CM codes for diastolic heart failure (I50.3) instead of the Desai et al. algorithm to identify individuals with HFpEF yielded similar results—systemic inflammation was significantly associated with a 32% increased risk of the composite HF endpoint (HR 1.32, 95% CI 1.12–1.55, *P* < 0.01) (Supplementary Table S2).

## 4 Discussion

This analysis represents the first large real-world observational study to characterize the demographics and outcomes of individuals with HFpEF with and without systemic inflammation. Systemic inflammation was associated with a 21% increase in the risk of a composite HF outcome, and also with statistically significant increases in the risk of subsequent HF hospitalization and urgent HF visits. This information adds to our understanding of the clinical burden of HFpEF, where therapies remain limited and the impact of cardiovascular inflammation is unclear.

The observed association between systemic inflammation and a higher risk of HF-related clinical outcomes is consistent with previous literature, despite variations in study populations, clinical endpoints, and methodologies. A *post-hoc* analysis of the TOPCAT (Aldosterone Antagonist Therapy for Adults with Heart Failure and Preserved Systolic Function) trial population found a high prevalence of systemic inflammation (62%, defined as hsCRP ≥2 mg/L) among individuals with HFpEF and that those with systemic inflammation appeared to have more frequent prior HF hospitalization, chronic obstructive pulmonary disease, and higher body mass index (10). The TOPCAT population with systemic inflammation also had a significantly higher risk of experiencing a composite event of cardiovascular death, resuscitated cardiac arrest, or HF hospitalization compared to those without systemic inflammation (adjusted HR 2.36, 95% CI 1.27–4.38).

As with our findings, systemic inflammation was not significantly associated with an increased risk of all-cause mortality in the TOPCAT analysis (adjusted HR 1.40, 95% CI 0.74–2.66) (10), suggesting that inflammation may have a greater impact on morbidity than mortality in HFpEF. Notably, the lack of association between SI and all-cause mortality in our study may be due, in part, to the relatively short average follow-up of approximately 33 months, which may have been insufficient to observe a mortality difference between the two cohorts. Additionally, a biological distinction between morbidity and mortality in HFpEF may explain these findings. Systemic inflammation in HFpEF is thought to promote myocardial remodeling, fibrosis, and impaired relaxation, leading primarily to diastolic dysfunction, congestion, and symptomatic decompensation that manifest as HF hospitalizations and urgent care visits. In contrast, mortality in HFpEF is influenced by a broader range of cardiovascular and non-cardiovascular factors, including arrhythmias, progressive HF, renal dysfunction, infection, and malignancy. A systematic review and meta-analysis of individuals with HFpEF demonstrated that elevated CRP was associated with new onset of HFpEF and subsequent increased risk of cardiovascular mortality (HR 2.52, 95% CI 1.61–3.96) and all-cause mortality (HR 1.78; 95% CI 1.53–2.06) (16). Although most studies in cardiovascular disease have utilized hsCRP as a marker of systemic inflammation, other inflammatory markers have been examined and associated with significantly increased risk for mortality and cardiovascular rehospitalization in HFpEF (17).

Notably, this study expands the existing literature on cardiovascular inflammation in individuals with HFpEF by utilizing a large and representative set of real-world administrative claims and laboratory data from the US. Despite differences in absolute values, we observed a similar clinical burden for HF compared to prior studies. However, a key distinction of the present study is the use of a real-world population and the inclusion of over 10,000 individuals with eligible hsCRP measurements. Our study highlights the existing care burden outside of the clinical trial environment and evaluates a substantially larger HFpEF population than prior analyses, e.g., 232 individuals with hsCRP values were included in the TOPCAT *post-hoc* study (10).

Among individuals with HF in our dataset, only 8.5% were tested for hsCRP throughout the study period from 2016 to 2023. Of those who were tested and had results available in the database, nearly half showed evidence of systemic inflammation. Increasing hsCRP testing among individuals with HF represents a potential opportunity to identify people at higher risk and to optimize therapy. The proportion of individuals with systemic inflammation in this study highlights the potential impact of therapies that target inflammation. Currently, there are no guideline recommendations supporting routine hsCRP or inflammatory biomarker testing in the evaluation of individuals with HF. Even in ASCVD, where elevated hsCRP is recognized as a risk enhancing factor by the American Heart Association and American College of Cardiology (18), hsCRP testing remains low (19). This suggests that the clinical burden associated with systemic inflammation is under-recognized, and broader hsCRP testing could enhance awareness of the unmet need associated with systemic inflammation in the HF pathophysiology.

Guideline-directed medical therapy for HFpEF remains limited. Recent breakthroughs from the EMPEROR-Preserved (20) and DELIVER trials (21) showed that SGLT2 inhibitors, including dapagliflozin and empagliflozin, significantly reduce cardiovascular death and heart failure hospitalizations in individuals with HFmrEF or HFpEF. The pathophysiological role of systemic inflammation in HFpEF makes it a potential therapeutic target (22) and both observational and trial evidence support the role of inflammation in the pathophysiology of HF (23, 24). There have been signals of the potential utility of anti-inflammatory therapies (25, 26). In the CANTOS trial, the interleukin-1β inhibitor canakinumab showed a dose-dependent reduction in HF hospitalization (27), while anakinra, an interleukin-1 receptor antagonist, lowered hsCRP and the myocardial stress marker N-terminal pro-B-type natriuretic peptide (NT-proBNP) but did not enhance exercise capacity in individuals with HFpEF (D-HART pilot study) (28).

This study is the first to examine the impact of systemic inflammation in HFpEF using real-world data. The study includes a large sample size but differs from clinical trial populations and introduces limitations associated with the identification of individuals with HFpEF from administrative claims data. Because LVEF data were limited, the Desai et al. algorithm identified individuals with HFpEF based on their clinical characteristics, HF diagnosis, treatments, and procedures. With a PPV of 0.84, approximately 1,760 (16%) patients may have been misclassified as having HFpEF when they actually have HFrEF. A sensitivity analysis that used only the ICD-10-CM code for diastolic HF to identify HFpEF yielded results similar to the primary analysis, supporting the validity of the algorithm-based approach. The demographics of the study population, which included individuals with a mean age of 66 years with underlying hypertension, dyslipidemia, and obesity, also align with previous epidemiologic studies of HFpEF (29). This supports the appropriateness of the cohort identification criteria. Additional limitations of the study include the degree of generalizability to broader populations and potential selection bias. The study was limited to individuals with HF who were tested for hsCRP and had the data available, which could differ from the general HF population. NT-proBNP is an established prognostic marker for HF outcomes (30), but NT-proBNP data reflecting HF severity were limited in this study (small sample size) and insufficient for inclusion in multivariable models. The absence of NT-proBNP adjustment may lead to residual confounding, highlighting the need for future research when data sources with sufficent capture are available.

All-cause mortality data in this study were derived from multiple administrative sources, including the Social Security Administration Death Master File, inpatient discharge status, and third-party obituary data. These sources, while comprehensive, may not capture all deaths. No evidence was observed to suggest that such underestimation was systematically different between patients with and without systemic inflammation. Finally, the study was observational by nature, and while it demonstrates the prognostic value of hsCRP, it does not establish a causal relationship.

Overall, this study highlights that the burden of systemic inflammation in HFpEF is underrecognized, extensive, and clinically meaningful. The results suggest that increased screening for systemic inflammation in HFpEF could help identify the individuals with HFpEF who are at the highest risk and in need of further treatment optimization to improve clinical outcomes.

## Data Availability

The original contributions presented in the study are included in the article/Supplementary Material, further inquiries can be directed to the corresponding author.
