# Supplementary material for: Systemic inflammation and the risk of adverse clinical outcomes in heart failure with preserved ejection fraction
Source: Front Cardiovasc Med. 2026 Aug 28;13:1872310. doi: 10.3389/fcvm.2026.1872310 (PMC13563271; doi:10.3389/fcvm.2026.1872310)
Supplement: Supplementary file 1 [file Table1.docx]

Supplementary Material

# 1 Supplementary Figures and Tables

## 2 Supplementary Tables

Supplementary Table s1. Representativeness of the study population by baseline characteristics

| Variables | Study population | Individuals with HFpEF after applying exclusion criteria | Individuals with HFpEF not tested for hsCRP | Individuals with HFpEF tested for hsCRP |
| --- | --- | --- | --- | --- |
| N | 11,015 | 2,181,247 | 1,995,610 | 185,637 |
| Age at index date |  |  |  |  |
| Mean (SD) | 66.4 (12.4) | 67.3 (13.7) | 67.5 (13.7) | 64.5 (13.4) |
| Range | 18.0 - 88.0 | 18.0 - 88.0 | 18.0 - 88.0 | 18.0 - 88.0 |
| Median (Q1, Q3) | 67.0 (58.0, 77.0) | 69.0 (58.0, 80.0) | 69.0 (59., 80.0) | 64.0 (56.0, 75.0) |
| Categorical age |  |  |  |  |
| 18-44 | 554 (5.0%) | 139,333 (6.4%) | 125,298 (6.3%) | 14,035 (7.6%) |
| 45-54 | 1,223 (11.1%) | 237,072 (10.9%) | 210,970 (10.6%) | 26,102 (14.1%) |
| 55-64 | 3,039 (27.6%) | 524,471 (24.0%) | 470,067 (23.6%) | 54,404 (29.3%) |
| 65-74 | 2,875 (26.1%) | 480,575 (22.0%) | 439,089 (22.0%) | 41,486 (22.4%) |
| 75-84 | 2,986 (27.1%) | 672,860 (30.9%) | 630,306 (31.6%) | 42,554 (22.9%) |
| ≥85 | 338 (3.1%) | 126,936 (5.8%) | 119,880 (6.0%) | 7,056 (3.8%) |
| Gender |  |  |  |  |
| Female | 5,362 (48.7%) | 1,156,718 (53.0%) | 1,058,680 (53.1%) | 98,038 (52.8%) |
| Male | 5,307 (48.2%) | 985,841 (45.2%) | 902,379 (45.2%) | 83,462 (45.0%) |
| Missing | 346 (3.1%) | 38,688 (1.8%) | 34,551 (1.7%) | 4,137 (2.2%) |
| Race/Ethnicity |  |  |  |  |
| White | 5,841 (53.0%) | 1,197,012 (54.9%) | 1,104,729 (55.4%) | 92,283 (49.7%) |
| Hispanic or Latino | 1,950 (17.7%) | 308,475 (14.1%) | 274,717 (13.8%) | 33,758 (18.2%) |
| Black or African   American | 1,495 (13.6%) | 368,554 (16.9%) | 336,579 (16.9%) | 31,975 (17.2%) |
| Asian or Pacific   Islander | 531 (4.8%) | 72,092 (3.3%) | 65,313 (3.3%) | 6,779 (3.7%) |
| Other | 455 (4.1%) | 65,328 (3.0%) | 58,543 (2.9%) | 6,785 (3.7%) |
| Missing | 743 (6.8%) | 169,786 (7.8%) | 155,729 (7.8%) | 14,057 (7.6%) |
| Region |  |  |  |  |
| Northeast | 3,054 (27.7%) | 505,073 (23.2%) | 452,713 (22.7%) | 52,360 (28.2%) |
| North Central | 1,234 (11.2%) | 485,828 (22.3%) | 455,950 (22.9%) | 29,878 (16.1%) |
| South | 3,593 (32.6%) | 752,151 (34.5%) | 697,181 (34.9%) | 54,970 (29.6%) |
| West | 3,123 (28.4%) | 410,210 (18.8%) | 363,771 (18.2%) | 46,439 (25.0%) |
| Missing | 11 (0.1%) | 27,985 (1.3%) | 25,995 (1.3%) | 1,990 (1.1%) |
| Insurance |  |  |  |  |
| Commercial | 3,473 (31.5%) | 479,189 (22.0%) | 428,053 (21.4%) | 51,136 (27.6%) |
| Medicaid | 1,384 (12.6%) | 404,950 (18.6%) | 364,342 (18.3%) | 40,608 (21.9%) |
| Medicare | 6,153 (55.9%) | 1,294,082 (59.3%) | 1,200,402 (60.2%) | 93,680 (50.5%) |
| Missing | 5 (0.1%) | 3,026 (0.1%) | 2,813 (0.1%) | 213 (0.1%) |
| QCI |  |  |  |  |
| Mean (SD) | 1.01 (1.3) | 1.19 (1.4) | 1.18 (1.4) | 1.23(1.4) |
| Categorical QCI |  |  |  |  |
| 0 | 5,303 (48.1%) | 926,881 (42.5%) | 852,127 (42.7%) | 74,754 (40.27%) |
| 1-2 | 4,315 (39.2%) | 899,662 (41.3%) | 819,733 (41.1%) | 79,929 (43.06%) |
| 3-4 | 1,211 (11.0%) | 294,310 (13.5%) | 268,519 (13.5%) | 25,791 (13.89%) |
| 5+ | 186 (1.7%) | 60,394 (2.8%) | 55,231 (2.8%) | 5163 (2.78%) |

**Abbreviations**: HFpEF, heart failure with preserved ejection fraction; hsCRP, high-sensitivity C-reactive protein; Q, quartile; QCI, Quan-Charlson Comorbidity Index

Supplementary Table s2. Sensitivity analysis of the HF composite endpoint using ICD-10-CM codes to identify individuals with HFpEF instead of the Desai’s algorithm (N=3,450)

| **Variable** | **HF composite endpoint** | |
| --- | --- | --- |
|  | **HR (95% CI)** | **P value** |
| **Systemic inflammation** |  |  |
| No | Reference |  |
| Yes | 1.32 (1.12, 1.55) | <0.01 |
| **Age** |  |  |
| 18-44 | Reference |  |
| 45-54 | 1.29 (0.65, 2.54) | 0.46 |
| 55-64 | 1.24 (0.66, 2.35) | 0.5 |
| 65-74 | 1.13 (0.58, 2.2) | 0.73 |
| 75-84 | 1.73 (0.88, 3.38) | 0.11 |
| ≥85 | 2.02 (0.95, 4.31) | 0.07 |
| **Gender** |  |  |
| Female | Reference |  |
| Male | 1.02 (0.86, 1.21) | 0.84 |
| **Race/Ethnicity** |  |  |
| White | Reference |  |
| Hispanic or Latino | 0.74 (0.58, 0.94) | 0.01 |
| Black or African American | 0.89 (0.69, 1.14) | 0.37 |
| Asian or Pacific Islander | 0.77 (0.51, 1.17) | 0.23 |
| Other | 0.83 (0.51, 1.35) | 0.45 |
| **Region** |  |  |
| North East | Reference |  |
| North Central | 1.13 (0.87, 1.48) | 0.36 |
| South | 1.02 (0.82, 1.27) | 0.85 |
| West | 1.26 (1.01, 1.57) | 0.04 |
| **Insurance** |  |  |
| Commercial | Reference |  |
| Medicaid | 1 (0.72, 1.39) | 0.99 |
| Medicare | 1.15 (0.86, 1.55) | 0.34 |
| **QCI** |  |  |
| 0 | Reference |  |
| 1-2 | 1.43 (1.18, 1.72) | <0.01 |
| 3-4 | 1.47 (1.13, 1.92) | <0.01 |
| 5+ | 1.06 (0.59, 1.89) | 0.85 |
| **Baseline conditions** |  |  |
| Atrial fibrillation | 1.21 (0.98, 1.49) | 0.08 |
| Depression | 1.06 (0.84, 1.33) | 0.62 |
| Dyslipidemia | 0.91 (0.75, 1.1) | 0.33 |
| Stroke | 1.47 (1.06, 2.03) | 0.02 |
| Hypertension | 1.3 (1, 1.69) | 0.05 |
| Hospitalization during 6 months prior to the index date | 1.07 (0.85, 1.35) | 0.56 |
| **Baseline medication** |  |  |
| Anti-inflammatory and immunosuppressant medications | 0.94 (0.69, 1.29) | 0.7 |
| ACE inhibitors | 0.99 (0.81, 1.2) | 0.89 |
| MRAs | 1 (0.69, 1.46) | 0.99 |
| ARBs | 0.82 (0.67, 1.01) | 0.06 |
| HF beta blockers | 1.19 (1, 1.42) | 0.06 |
| Other beta blockers | 1.3 (1.03, 1.64) | 0.03 |
| Loop diuretics | 1.86 (1.55, 2.24) | <0.01 |
| Other diuretics | 0.77 (0.58, 1.03) | 0.08 |
| Hydralazine/nitrate combination | 1.39 (1.06, 1.81) | 0.02 |
| Digoxin | 1.39 (0.78, 2.47) | 0.27 |
| Sacubitril/valsartan or ivabradine | NA | NA |
| SGLT2 inhibitors | 1.43 (0.96, 2.14) | 0.08 |

Abbreviations: ACE, angiotensin converting enzyme; ARB, angiotensin receptor blocker; CI, confidence interval; HF, heart failure; HR, hazard ratio; MRA, mineralocorticoid receptor antagonist; QCI, Quan-Charlson Comorbidity Index; SGLT2, sodium-glucose cotransporter-2
